# Anti-Inflammatory Effects of Berberine Hydrochloride in an LPS-Induced Murine Model of Mastitis

**DOI:** 10.1155/2018/5164314

**Published:** 2018-04-15

**Authors:** Xichun Wang, Shibin Feng, Nana Ding, Yanting He, Cheng Li, Manman Li, Xuedong Ding, Hongyan Ding, Jinchun Li, Jinjie Wu, Yu Li

**Affiliations:** College of Animal Science and Technology, Anhui Agricultural University, 130 West Changjiang Road, Hefei 230036, China

## Abstract

Berberine hydrochloride is an isoquinoline type alkaloid extracted from Berberidaceae, Rutaceae, and other plants. Previous reports have shown that berberine hydrochloride has anti-inflammatory properties. However, the underlying molecular mechanisms remain unclear. In this study, a lipopolysaccharide- (LPS-) induced murine model of mastitis was established to explore the anti-inflammatory action of berberine hydrochloride. Sixty mice that had been lactating for 5–7 days were randomly divided into six groups, including control, LPS, three berberine hydrochloride treatment groups (5, 10, and 20 mg/kg), and a dexamethasone (DEX) (5 mg/kg) group. Berberine hydrochloride was administered intraperitoneally 1 h before and 12 h after LPS-induced mastitis, and all mice were sacrificed 24 h after LPS induction. The pathological and histopathological changes of the mammary glands were observed. The concentrations and mRNA expressions of TNF-*α*, IL-1*β*, and IL-6 were measured by ELISA and qRT-PCR. The activation of TLR4 and NF-*κ*B signaling pathways was analyzed by Western blot. Results indicated that berberine hydrochloride significantly attenuated neutrophil infiltration and dose-dependently decreased the secretion and mRNA expressions of TNF-*α*, IL-1*β*, and IL-6 within a certain range. Furthermore, berberine hydrochloride suppressed LPS-induced TLR4 and NF-*κ*B p65 activation and the phosphorylation of I-*κ*B. Berberine hydrochloride can provide mice robust protection from LPS-induced mastitis, potentially via the TLR4 and NF-*κ*B pathway.

## 1. Introduction

**Figure 1:**
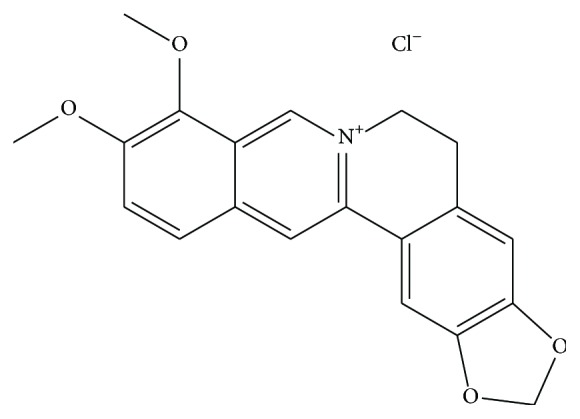
The chemical structure of berberine hydrochloride.

Inflammation is a complex reaction that occurs within local or systemic animal organs in response to multiple endogenous or exogenous injuries, which mainly result in redness, swelling, fever, pain of organs, and tissue damage [1]. Berberine hydrochloride (C_20_H_19_CLNO_4_, Figure 1), also known as berberine, is a quaternary ammonium compound among Rhizoma Coptidis, Cortex Phellodendri, and other plants, and is found in Berberidaceae, Ranunculaceae, Rutaceae, and other plants containing isoquinoline alkaloid [2]. Moreover, related studies have demonstrated berberine hydrochloride has the effect of anti-inflammatory, lipid-lowering, hypoglycemic, immunoregulatory, hepatoprotective, and anticancer activity [3–6]. In addition, there have been multiple reports regarding the anti-inflammatory effects of berberine hydrochloride, such as Zhang et al., who found that berberine hydrochloride had a significant protective effect on mice liver injury induced by LPS and can reduce the secretion of IL-1 and TNF-*α* [7]. Moreover, Nemoto et al. confirmed that berberine hydrochloride exhibits a positive effect on the prevention of postoperative intestinal adhesion and inflammation in mice, can markedly reduce the density of adhesion and severity, and also significantly reduces the expression level of corresponding inflammatory mediators [8]. Although multiple anti-inflammatory and pharmacological effects of berberine hydrochloride have been widely recognized throughout the world, the underlying molecular mechanisms of LPS-induced murine mastitis have yet to be reported systematically. The pathogenic microorganisms invade the breast epithelial cells or breast tissue loss; they will cause breast cancer defensive inflammatory response. Early in the inflammatory response, vasodilation occurs in impaired breast areas, vascular permeability increases, and blood vessels have inflammatory mediators (histamine, bacterial toxins, and leukotrienes) and other symptoms. As the exudate contains coagulation factors, it will cause lactation disorders. If breast stimulation stops in a short period of time, the inflammation in the blood vessels will decrease accordingly. If the infection is more serious, it will cause damage to the secretion of acinar, so that the lactation function of the lactation area is lost [9].

The main pathogenic factor of* Escherichia coli* is lipopolysaccharide (LPS), which is the main component of the bacterial cell wall [10]. LPS is typically used to establish animal models of mastitis using the method of perfusing into the lactating mice's teat surgically inserting a container, and this method has been widely recognized [11]. Previous studies have found that the inflammatory response induced by LPS is primarily mediated by TLR4 receptor. TLR4 is activated; it can combine with the molecular chaperone heat shock protein 60, activating downstream nuclear factor *κ*B (NF-*κ*B), which finally induces macrophages to produce proinflammatory cytokines [12]. Such cytokine production is characterized by tumor necrosis factor *α* (TNF-*α*), interleukin-1*β* (IL-1*β*), and interleukin-6 (IL-6), which induces neutrophil recruitment, an inflammatory reaction, and tissue damage [13, 14]. Penicillin, streptomycin, and ciprofloxacin are commonly used drugs for the treatment of mastitis, but with the frequent use of drugs, drug resistance continues to increase, and its therapeutic effect decreased [15]. Recently, inhibiting the inflammatory response via targeting signal transduction pathways has become a novel approach for the treatment of severe inflammation.

In this study, we used the LPS/TLR4/NF-*κ*B signal transduction pathway as a theoretical mode to explore (1) the anti-inflammatory effect of berberine hydrochloride on mastitis in mice induced by LPS and (2) the associated molecular mechanisms.

## 2. Material and Methods

### 2.1. Animals

A total of 60 female and 20 male BALB/c mice (6–8 weeks old, weighing 35–40 g) were purchased from Changzhou Covance Experimental Animal limited company (Changzhou, China). The female and male mice cohabited at a 3 females to 1 male ratio for conception. They were housed separately after pregnancy until five to seven days after calving. Lactating and female mice were raised in a room maintained at 24°C ± 1°C with 40%–80% humidity and were allowed access to food and water ad libitum.

### 2.2. Reagents

Berberine hydrochloride standard (Control of Pharmaceutical and Biological Product, Beijing, China) and LPS (Escherichia coli 055:B5) were purchased from Sigma Chemical Co. (St. Louis, MO, USA); Mouse TNF-*α*, IL-6, and IL-1*β* ELISA kits were purchased from Senbeijia Biological Technology Co. (Nanjing, China). The primary monoclonal antibodies (mAbs), anti-mouse TLR4, anti-mouse p65, and anti-mouse phospho-p65 monoclonal antibodies were purchased from Santa Cruz Biotechnology (CA, USA). The primary anti-rabbit I*κ*B*α*, anti-mouse phospho-I*κ*B*α* antibodies, and the secondary goat anti-rabbit IgG and goat anti-mouse IgG antibodies were purchased from Beyotime Biotechnology Co. (Nanjing, China).

### 2.3. The Mouse Model of Mastitis and Experimental Design

Approximately five to seven days after delivery, the lactating mice were separated from the offspring 1 h before the experiment. The female mice were infused with sodium pentobarbital through the abdominal cavity using a 1 mL syringe to induce anesthesia; 2-3 min later, the mice were placed in a supine position under the stereomicroscope. There are five pairs of mammary glands (MGs) in the mice and the fourth pair is usually used to establish mastitis because it is the largest of the five and is the easiest to observe and harvest. The fourth mammary gland and surrounding skin were disinfected with a cotton ball soaked in 75% alcohol to fully expose the breast. The nipple was fixed in the left hand with tweezers, and the tip of the nipple was cut 1 mm with sterile ophthalmic to expose the milk ducts. A 100 *μ*L microinjector with a 30 G blunt needle was slowly inserted into the milk ducts using the right hand, and 50 *μ*L of a 0.2 mg/mL LPS solution was injected into the mammary gland for a few seconds before pulling the needle out.

### 2.4. Test Groups and Methods of Administration

The lactating mice were randomly divided into six groups (10 mice/group): a blank control group, LPS (10 *μ*L/a mouse) group, berberine hydrochloride (5 mg/kg, 10 mg/kg, and 20 mg/kg) + LPS group, and a dexamethasone (DEX) (5 mg/kg) + LPS group. Berberine hydrochloride and DEX were administered 1 h before and 12 h after the LPS infusion through the abdominal cavity. The mice from LPS and control groups were given the same volume of PBS. All mice were euthanized 24 h after the LPS or PBS injection, and the breast tissue was collected for follow-up analysis.

### 2.5. Histopathologic Evaluation of the Breast Tissue

Fresh breast gland tissue samples from each treatment group were collected and fixed in a 10% formalin solution for approximately 48–72 h, then embedded in paraffin, sectioned, and stained by hematoxylin and eosin (H&E). Each experiment was repeated three times. The pathological changes were observed by a light microscopy (Olympus, Japan).

### 2.6. Measure of TNF-*α*, IL-1*β*, and IL-6 Levels in the Breast Tissue

The mammary gland tissues of the mice were mixed with PBS (1 : 9, w/v, g/mL) followed by homogenization and centrifugation 2000*g* at 4°C for 5 min. The supernatant was collected with a new centrifuge tube. The level of TNF-*α*, IL-1*β*, and IL-6 in the breast tissue was detected using commercial ELISA kits, according to the manufacturer's instructions (Sbjbio Company, Nanjing, China). Each experiment was repeated three times.

### 2.7. Total RNA Isolation from Breast Tissue and qRT-PCR

The mammary gland tissue of the mice was collected and stored at −80°C. The tissue (50 mg tissue) was ground into a powder at the time of detection, and the total RNA was extracted using Trizol reagent following the manufacturer's instructions (TaKaRa, Dalian, China). The RNA was reverse-transcribed into cDNA in accordance with the Reverse Transcription System's instructions (TaKaRa, Dalian, China). Reactions were performed in a 25-*μ*l reaction mixture containing 12 *μ*l of 2x SYBR Green I PCR Master Mix (TaKaRa, China), 2 *μ*l of diluted cDNA, 0.5 *μ*l of each primer (10 *μ*M), 0.8 *μ*l of 50x ROX reference Dye II, and 9.2 *μ*l of PCR-grade water. The PCR procedure for TNF-*α*, IL-1*β*, IL-6, and *β*-actin consisted of 95°C for 30 s, followed by 35 cycles of 95°C for 15 s, 63°C for 30 s, and 60°C for 30 s. A melting curve analysis showed only one peak for each PCR product. A dissociation curve was run for each plate to confirm the production of a single product. According to the GenBank sequence, the primer sequence of the target genes (i.e., TNF-*α*, IL-1*β*, and IL-6) and the *β*-actin gene were designed using the software Primer Premier 5.0 (Table 1). The amplification products were analyzed by 1.5% agarose gel electrophoresis and a gel imaging and analysis system (UVItec, Cambridge, UK). The mRNA relative abundance was calculated according to the method of Pfaffl and was normalized to the mean expression of *β*-actin. Results (fold changes) were expressed as 2^−ΔΔCt^ [16]:

(1) ΔΔCt=CtTNF-α/IL-1β/IL-6−Ctβ-actint−CtTNF-α/IL-1β/IL-6−Ctβ-actinc.

where t is the treatment group and c is the control group.

Ct_TNF-*α*/IL-1*β*/IL-6_ and Ct_*β*-actin_ are the cycle thresholds for TNF-*α*/IL-1*β*/IL-6 and *β*-actin genes in the different treated groups, respectively.

### 2.8. Western Blot Analysis of the Protein Expression of TLR4 and the NF-*κ*B Pathway

The mixture of protein lysate, proteinase inhibitor, and protein phosphatase inhibitor was added to the weighted mice mammary gland tissue in a proportion of 1 : 9 (w/v, g/mL). The fourth mammary gland tissue from each treatment was then ground into a homogenate on ice, and the centrifuged supernatant and lower residue were discarded and the supernatant was collected into a new centrifuge tube. The total protein concentration was determined using a BCA Protein Assay Kit (Beyotime Biotechnology Co., Shanghai, China). The protein (50 *μ*g) was split, and the SDS-PAGE loading buffer (Beyotime Biotechnology Co., Shanghai, China) was added, then placed into a 100°C water bath for 10 min, and preserved at 4°C for later use.

The gel board was installed, a 12% separating gel and 5% stacking gel were produced, and the comb was inserted. The comb was removed after coagulation and five protein samples were added to the SDS-PAGE electrophoresis. Electrophoresis was initiated at 80 V, changed to 120 V when the sample reached the two plastic boundaries of the transfer voltage, and continued until the samples ran to the bottom of the gel. The desired gel was cut according to the molecular weight indicated by the marker, and the band was transferred onto the polyvinylidene difluoride (PVDF) membrane using a semidry transfer membrane solution. The membranes were incubated in a 5% solution of bovine serum albumin (BSA) for 4 h at room temperature and then incubated with the primary antibody overnight at 4°C. The next day, the membrane was washed with TBST (Tris Buffered Saline, with Tween-20) three times, shaken for 5 min on the horizontal shaker, and then incubated with the secondary antibody at room temperature for 45 min on the horizontal shaker. The blots were washed three times with TBST and shaken for 5 min on the horizontal shaker. Finally, the blots were imaged using a Gel Imaging System (Bio-Rad, Hercules, CA, USA) and results were analyzed with Quantity One Software (Bio-Rad, Hercules, CA, USA). The *β*-actin protein was used as internal control.

### 2.9. Statistical Analysis

All data are presented as the mean ± SEM. Differences between the mean values of the normally distributed data were assessed by a one-way analysis of variance (ANOVA) (Dunnett's *t-*test) and a two-tailed Student's *t-*test. The criterion for the differences was considered significant at *P* < 0.05 in all studies.

## 3. Results

### 3.1. Effect of Berberine Hydrochloride on the Histopathological Changes of the Mammary Glands Induced by LPS in Mice with Mastitis

**Figure 2:**
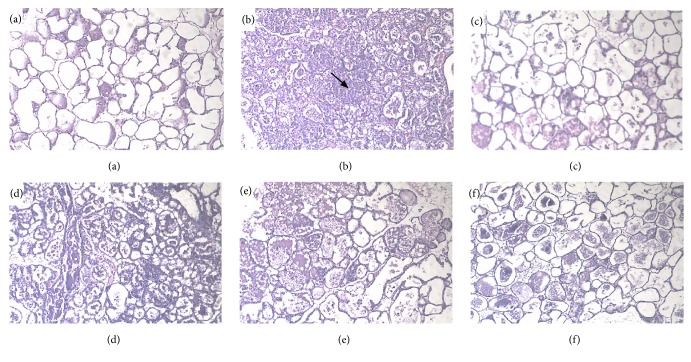
Histopathological sections of mouse mammary glands (hematoxylin and eosin staining, ×400).* Note*. (a) control group; (b) LPS group; (c) LPS + berberine hydrochloride (5 mg/kg) group; (d) LPS + berberine hydrochloride (10 mg/kg) group; (e) LPS + berberine hydrochloride (20 mg/kg); (f) LPS + DEX group. The arrow refers to the area of inflammatory infiltration in mammary gland.

Pathological sections of the breast tissue are presented in Figure 2. The breast tissue of the control group displayed a normal breast alveolar structure and no histopathologic changes (Figure 2(a)). In contrast, the breast tissue of the LPS group exhibited characteristic histopathologic changes, which included areas of inflammatory infiltration and exfoliated cellular components, and the mammary alveolar walls were thickened and congested (Figure 2(b)). However, the LPS-induced pathological changes were attenuated by berberine hydrochloride or DEX in a dose-dependent manner (Figures 2(c)–2(f)). In addition, the leakage of neutrophils, mammary alveolar detachment, and acinar wall edema were reduced. Moreover, breast tissue congestion and edema were also relieved.

### 3.2. Effects of Berberine Hydrochloride on TNF-*α*, IL-1*β*, and IL-6 Levels in the Mammary Glands of LPS-Induced Mice with Mastitis

**Figure 3:**
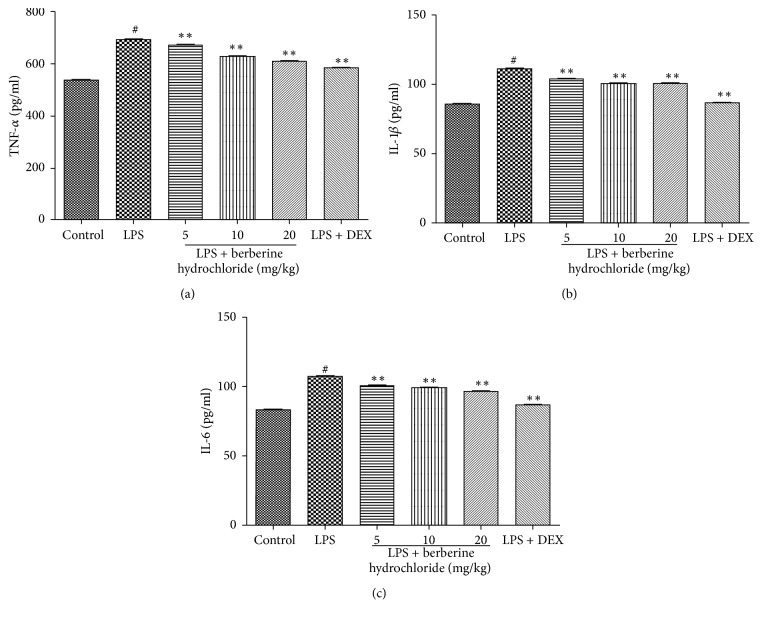
The production of TNF-*α*, IL-1*β*, and IL-6 in mouse mammary glands.* Note*. # indicates that blank group was compared to the LPS group (*P *< 0.01); *∗∗* indicates that the berberine hydrochloride group was compared to the LPS group (*P *< 0.01).

To explore the anti-inflammatory effects of berberine hydrochloride on LPS-induced endometritis, TNF-*α* and IL-1*β* levels in the mammary glands were detected by an ELISA. As presented in Figures 3(a)–3(c), the proinflammatory cytokine levels in the LPS group were significantly higher than that of the control group (*P *< 0.01). Moreover, the inflammatory cytokine levels of the berberine hydrochloride groups (doses of 5, 10, and 20 mg/kg) were significantly lower than the LPS group (*P* < 0.01) and gradually decreased with the increasing doses of berberine hydrochloride. The cytokine levels of the DEX group (5 mg/kg) were also significantly lower than the LPS group (*P* < 0.01).

### 3.3. Effects of Berberine Hydrochloride on TNF-*α*, IL-1*β*, and IL-6 mRNA Levels in the Mammary Glands of LPS-Induced Mice with Mastitis

**Figure 4:**
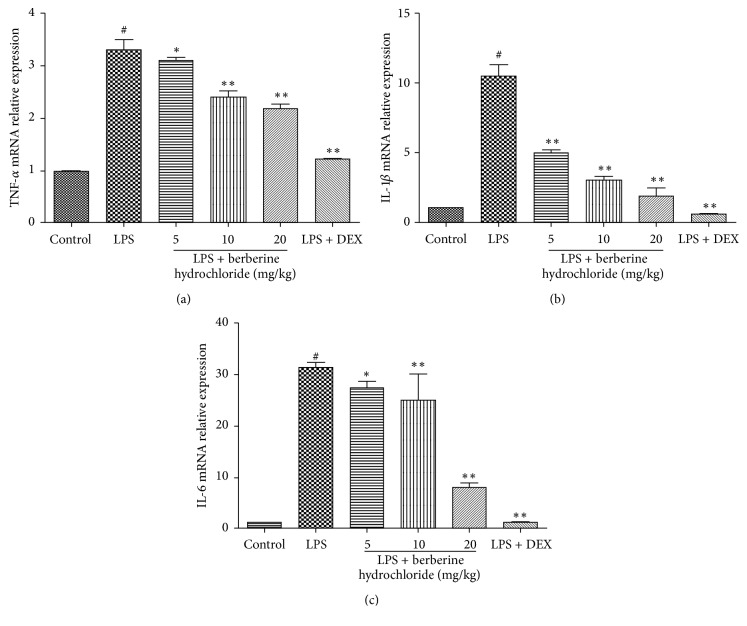
The mRNA production of TNF-*α*, IL-1*β*, and IL-6 in mouse mammary glands.* Note*. # indicates that the blank group was compared with the LPS group (*P *< 0.01); *∗* and *∗∗* indicate that the berberine hydrochloride group was compared with the LPS group (*P *< 0.05 and* P *< 0.01).

Next, we sought to evaluate the effects of berberine hydrochloride on the expression level of the proinflammatory cytokines by qRT-PCR. As shown in Figures 4(a)–4(c), the mRNA expression of the proinflammatory cytokines in the LPS group was significantly higher than that of the control group (*P* < 0.01). Moreover, the mRNA expression levels of the proinflammatory cytokines in the berberine hydrochloride groups (doses of 5, 10, and 20 mg/kg) were significantly lower than that of the LPS group (*P* < 0.05 or* P *< 0.01) and decreased in a dose-dependent manner. The cytokine mRNA levels of the DEX group (5 mg/kg) were also significantly lower than the LPS group (*P* < 0.01).

### 3.4. Effects of Berberine Hydrochloride on the TLR4 and NF-*κ*B Pathway in the Mammary Glands of LPS-Induced Mice with Mastitis

**Figure 5:**
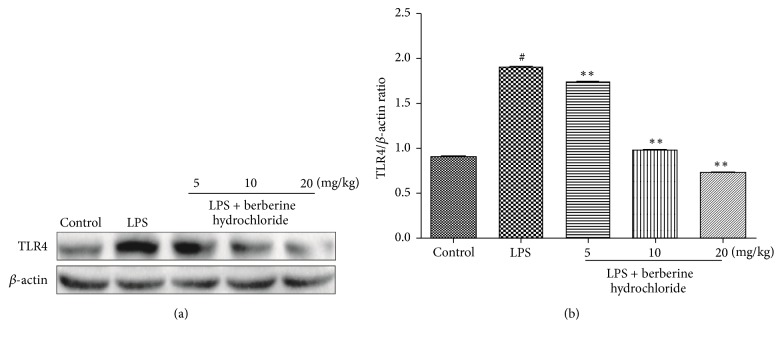
The protein production of TLR4 in mouse mammary glands.* Note*. # indicates that the blank group was compared to the LPS group (*P *< 0.01); *∗∗* indicates that the berberine hydrochloride group was compared to the LPS group (*P *< 0.01).

The effect of berberine hydrochloride on LPS-stimulated TLR4 protein expression levels in murine mastitis is shown in Figure 5. TLR4 protein expression was significantly increased following LPS stimulation, whereas berberine hydrochloride was significantly inhibited by increasing TLR4 protein following LPS stimulation in a dose-dependent manner. These findings indicate that berberine hydrochloride inhibits activation of the TLR4 pathway.

**Figure 6:**
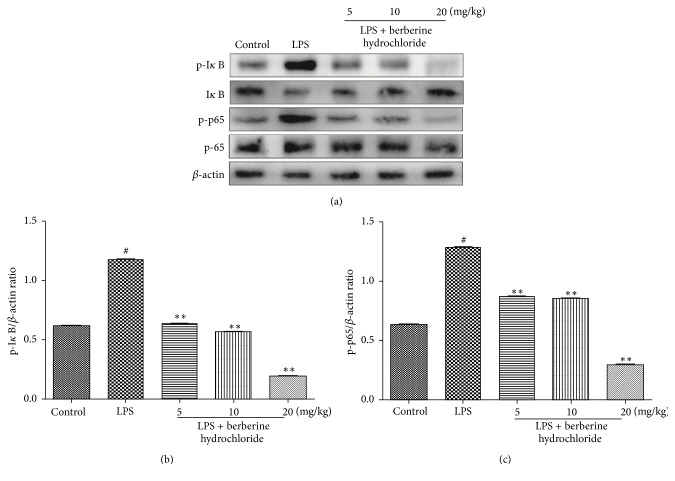
The protein expression of NF-*κ*B in the mouse mammary glands.* Note*. # indicates that the blank group was compared to the LPS group (*P *< 0.01); *∗∗* indicates that the berberine hydrochloride group was compared to the LPS group (*P *< 0.01).

Figure 6 shows the effects of berberine hydrochloride on LPS-stimulated NF-*κ*B protein expression in mice with mastitis. LPS stimulates the breast tissue to phosphorylate I*κ*B and subsequently release NF-*κ*B-p65 protein into the nucleus, thus inducing the production of proinflammatory cytokines. Following LPS stimulation of the breast tissue, the amount of phosphorylated I*κ*B increased significantly, the level of unphosphorylated I*κ*B significantly decreased, and phosphorylated NF-*κ*B-p65 also significantly increased. Compared to the LPS group, the levels of phosphorylated I*κ*B and phosphorylated NF-*κ*B-p65 were significantly decreased in the berberine hydrochloride groups (5, 10, and 20 mg/kg) in a dose-dependent manner. At the same time, the level of phosphorylated I*κ*B also increased as the drug concentration was elevated. These results indicate that berberine hydrochloride can inhibit the activation of the NF-*κ*B pathway.

## 4. Discussion

Berberine hydrochloride has been used clinically for several years as an antibacterial drug and also exhibits anti-heart failure [17], antiviral, and blood pressure lowering effects. Previous studies have confirmed that berberine hydrochloride has obvious protective effects on LPS-induced endometritis in mice. In particular, neutrophil infiltration was substantially reduced, the myeloperoxidase (MPO) concentrations were determined, and the anti-inflammatory mechanisms were preliminary confirmed to be through the activation of the NF-*κ*B signaling pathway [18]. However, the specific mechanism by which berberine hydrochloride is protective in LPS-induced mastitis in mice has not been previously reported. In this study, the anti-inflammatory effects of the traditional Chinese medicine, berberine hydrochloride, on LPS-induced murine mastitis were detected and analyzed in a variety of aspects; the results revealed that berberine hydrochloride exhibits a therapeutic effect on LPS-induced mastitis in mice.

When inflammation occurs in the mammary gland, the acinar will disassociate and degenerate, the breast tissue exhibits obvious congestion, and there is a large extent of lymphocyte infiltration in the mammary gland [19]. The results confirmed that the mouse breast exhibited obvious histopathological changes after LPS stimulation of the mammary gland, which could be significantly reduced by berberine hydrochloride in a dose-dependent manner within a specific range.

LPS can stimulate mammary epithelial cells to produce the inflammatory cytokines TNF-*α*, IL-1*β*, and IL-6, which can interact with and regulate the occurrence and outcome of mastitis [20]. TNF-*α* is primarily produced by monocytes or macrophages and can stimulate the production of other inflammatory cytokines (e.g., IL-1*β* and IL-6), induce neutrophil exudation, and promote oxygen free radical secretion, eventually resulting in breast tissue injury. IL-1*β* is an IL-1 subtype that can aggravate the inflammatory effects of TNF-*α*. IL-6 is involved in the inflammatory response of blood vessels and can regulate cellular proliferation and differentiation, the immune response, and stress response [21]. In this study, the content and gene expression of TNF-*α*, IL-1*β*, and IL-6 in the mammary gland tissues of LPS-stimulated mice were detected by an ELISA and qRT-PCR, respectively. The results indicate that berberine hydrochloride can significantly inhibit the increase of LPS-induced levels of cytokines and gene expression, revealing that berberine hydrochloride can relieve the inflammatory injury of mammary gland tissue induced by LPS.

To explore the mechanism of berberine hydrochloride in attenuating LPS-induced murine mastitis, we selected TLR4 and NF-*κ*B, two classical signal transduction pathways to examine. TLRs constitute an important class of pattern recognition receptors that recognize endogenous antigens of many microorganisms and play an important role in immune defense [22]. In particular, TLR4 is the main receptor recognizing LPS and is primarily expressed on monocytes, macrophages, epithelial cells, and neutrophils [23]. When TLR4 expressed on the cell membrane is stimulated by LPS, stimulatory signals are transduced downstream to activate NF-*κ*B. NF-*κ*B is an important transcription factor involved in immunity and inflammation and is widely found in mammalian cells [24]. Anti-inflammatory activities displayed by berberine may be mediated in part through the suppression of the NF-*κ*B activation pathway [25]. Under resting conditions, NF-*κ*B and I*κ*B are polymerized into a trimer within the cytoplasm. When the trimer is stimulated by LPS or other external factors, I*κ*B is phosphorylated and degraded, subsequently activating NF-*κ*B [26]. These may provide the molecular basis for the ability of berberine to act as an anti-inflammatory agent. The Western blot results in our study showed that LPS could activate the TLR4 and NF-*κ*B signal transduction pathway, and berberine hydrochloride could downregulate the phosphorylation of NF-*κ*B p65 and I*κ*B by inhibiting the expression of TLR4. Subsequently, the activation of the TLR4 and NF-*κ*B signaling pathways was inhibited, and the expression and secretion of proinflammatory cytokines were reduced. Finally, the damage sustained to the mammary gland tissue in response to LPS was alleviated.

## 5. Conclusions

The present study demonstrates that berberine hydrochloride exerts anti-inflammatory effects by inhibiting inflammatory cell infiltration, the production of TNF-*α*, IL-1*β*, and IL-6, and the activation of the TLR4/NF-*κ*B signaling pathway in a mouse model of mastitis. These results indicate that berberine hydrochloride might be a potential therapeutic agent against mastitis.

## Figures and Tables

**Table 1:** The primer sequences of TNF-*α*, IL-1*β*, and IL-6.

Gene	Primer	Sequence 5′→3′	Product size (bp)
*TNF-α*	Sense	GTCTCAGCCTCTTCTCATTC	128
	Antisense	CATAGAACTGATGAGAGGGA	
*IL-1β*	Sense	AAATACCTGTGGCCTTGGGC	101
	Antisense	CTTGGGATCCACACTCTCCAG	
*IL-6*	Sense	GAGTCCTTCAGAGAGATACAG	125
	Antisense	CTGTGACTCCAGCTTATCTG	
*β-actin*	Sense	CTTCATTGACCTCAACTACATGG	134
	Antisense	CTCGCTCCTGGAAGATGGTGAT	
